# The costs of saving nature: Does it make “cents”?

**DOI:** 10.1371/journal.pbio.2003292

**Published:** 2017-07-31

**Authors:** Andrew J. Tanentzap

**Affiliations:** Ecosystems and Global Change Group, Department of Plant Sciences, University of Cambridge, Cambridge, United Kingdom

## Abstract

Clearing wild forests to grow food, fibre, and fuel products can deliver large financial gains. However, the benefits that people obtain from forests—known as ecosystem services—are rarely considered in economic calculations, partly because there are few markets onto which they can be traded. In some regions, the benefits delivered by nature might be more economically valuable. A new study maps where it is profitable to replace tropical forests with cropland and how this might change under future agricultural production and carbon prices. The findings address a major applied challenge by helping to identify sites where forest conservation can be economically viable.

As you read this sentence, an area of forest equivalent to several soccer fields has been chopped down somewhere in the world [1]. With the loss of those trees, so too have the benefits disappeared that people obtained from that forest, such as carbon sequestration, water purification, and biodiversity [2]. And it is unlikely that such deforestation will slow any time in the near future given the need to provide additional food, fibre, and fuel for a growing human population. Forest clearance will likely be sustained even if yields are boosted on under-performing farmland [3,4], because it will take decades for yield increases to materialise. Regional policies, diminishing returns for additional farmland inputs, and variable access to capital, technology, and infrastructure all pose major obstacles to yield gains [4,5]. Taken with the relentless pace at which agriculture is expanding, particularly in the tropics [6–8], there may be little forest left unconverted by the time farmers fully close the worldwide gap between actual and maximum attainable yields.

## Making the business case for protecting forests

Incentivising people to avoid converting forest may offer at least a stopgap for slowing the global march of land-use change. The richest of these efforts is the United Nation’s initiative for Reducing Emissions from Deforestation and Forest Degradation (REDD+). But choosing which forests to protect is a major research challenge. Not only are biodiversity and ecosystem services distributed unevenly across the world’s forests [9] but replacing forest with agriculture can produce financial and ecosystem services in some places that offset the environmental costs of land conversion. Turning forests into a commodity, however, risks making conservation an economic decision with competing outcomes. In some cases, there may be a net economic loss if agriculture is foregone in favour of forest conservation—these decisions are broadly known as “opportunity costs” (Box 1).

**Box 1. What is an opportunity cost?**

**Fig 1:**
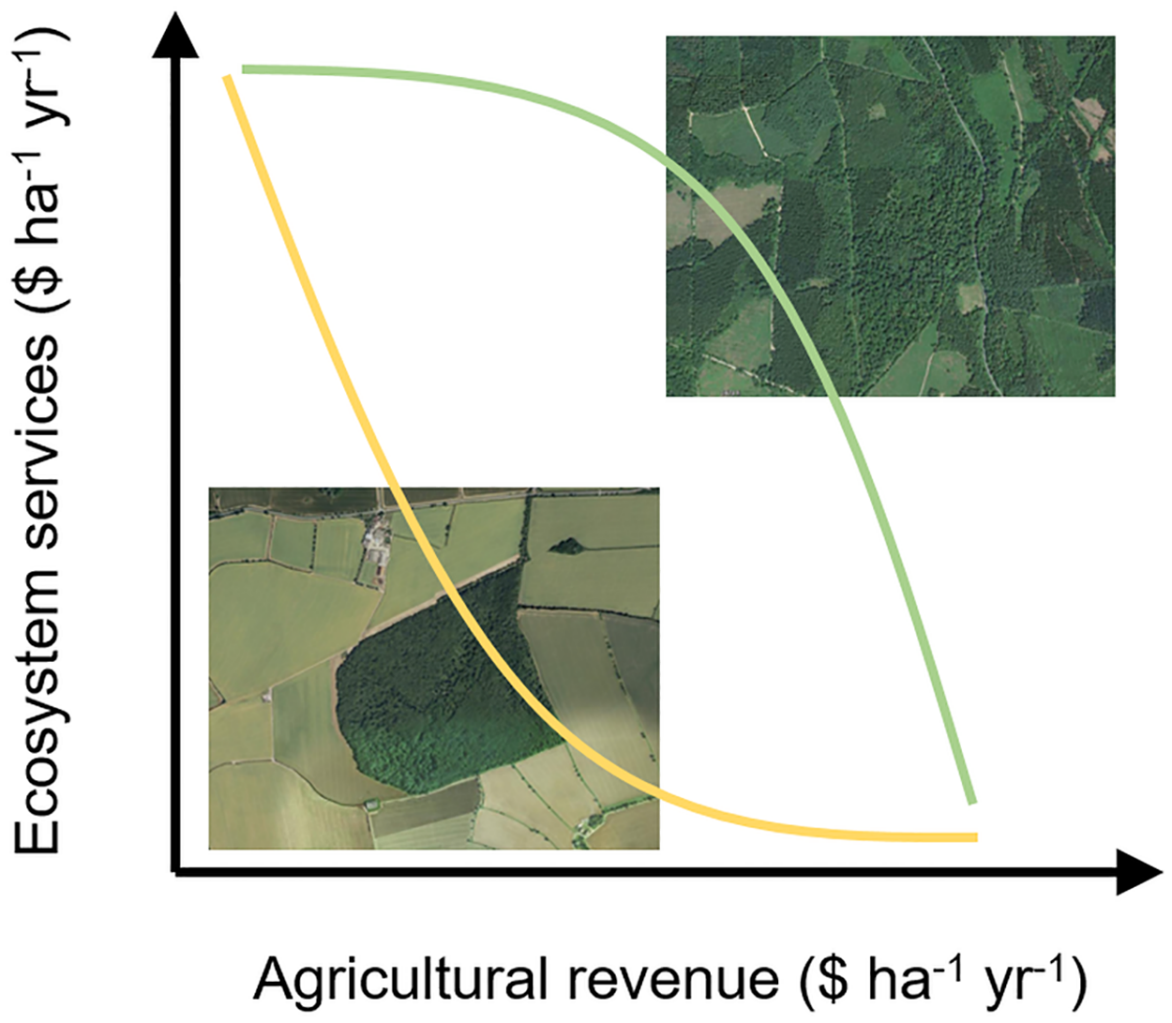
Hypothetical trade-offs between the value of ecosystem services and agricultural revenue in 2 landscapes with contrasting forest cover. Aerial images sourced from the LINZ Data Service (www.linz.govt.nz) and licensed for reuse under CC-BY 3.0.

Opportunity costs—the benefits lost by foregoing a course of action for a mutually exclusive alternative—are an economic concept that is central to quantifying the consequences of land-use decisions. In conservation, these costs are often the revenues, such as from agriculture or timber harvest, which were foregone by protecting wild nature [10]. Similarly, there are major opportunity costs associated with converting land from its natural state into agriculture, such as to carbon sequestration, water supplies, and recreational and cultural experiences. An entirely rational actor would make planning decisions that maximized net value, i.e., the value of a given land cover after accounting for the opportunity cost of alternative uses. But a major challenge is quantifying value in comparable terms across disparate benefits, for example, comparing crop yields with biodiversity. Monetary units are often used to express value but should not be the only approach that is considered. Spatial context will also mediate the strength of opportunity costs. As shown in Fig 1, one possible prediction is that revenue from agricultural production might trade off strongly against the value of ecosystem services in landscapes with little surrounding forest cover (shown by the yellow line). This trade-off would produce a greater opportunity cost to land conversion than where forest is much more abundant (shown by the green line in Fig 1). A major research opportunity is characterising the nature of this trade-off and the conditions that might mitigate it, such as through economies of scale in agriculture.

Given the opportunity costs that are involved with commodifying land-use decisions, it is critical that the services provided by forests are accurately quantified in biophysical and monetary units. But monetary valuation is notoriously difficult for 2 main reasons. First, valuing an ecosystem service is challenging when there are no markets on which it is directly traded [11]. In some cases, it may be possible to estimate monetary values from the costs that would be incurred if the ecosystem service had to be artificially recreated, such as through planting an entire forest for carbon sequestration (“cost-based methods”). For other ecosystem services, monetary value may be derived by directly asking people how much they are willing to pay for a given ecosystem service (i.e., “stated preference methods”). Monetary value can also be estimated from people’s willingness to pay for related goods or services, such as the change in property value from proximity to a given forest (i.e., “revealed preference methods”). The second challenge is transferring the values of services assessed at a given site to elsewhere because they will depend on socioeconomic and ecological context [12]. For example, the total value of flood control by an intact forest is higher when there are more people and infrastructure downstream [13]. Therefore, there is no one global estimate of the value of ecosystem services per hectare of forest.

Meta-analysis is one approach that can overcome some of the challenges associated with valuing ecosystem services under different land uses. By collating a diverse set of studies, statistical models can be developed that relate monetary values for ecosystem services to methodological variables (e.g., valuation methods), economic context (e.g., how easily humans benefit from ecosystem services), and ecological factors (e.g., climatic variables that influence levels of ecosystem services). These models can then be used to predict monetary values at entirely new sites given a set of assumptions and site-specific data. Efforts to collate valuation studies of ecosystem services into large databases [14,15] have made it readily possible to map the economic trade-offs between competing land uses.

## Global trade-offs in saving forests from agriculture

A new paper in this issue of *PLOS Biology* [16] tests how different scenarios of global agricultural production might trade off against the ecosystem services delivered by tropical forests. Identifying the spatial distribution of net economic losses and gains resulting from conversion of forest to agriculture will inform pantropical land-use policies and have considerable significance for global conservation efforts such as REDD+. In their study, Carrasco et al. [16] use the most comprehensive database of the monetary value of ecosystem services [15] to develop meta-analytical models that estimate the value of tropical forests at a 0.1° resolution. They combine these estimates with the costs of carbon emissions foregone from avoided deforestation and compare the values against potential agricultural yields on these same lands given recent (2000–2012) and potential crop distributions. A major advance is considering more ecosystem services than carbon stocks [17,18], such as the provisioning of food and raw materials, opportunities for recreation and tourism, and regulation of soils, climate, and water flows.

The main finding of Carrasco et al. [16] is that the value of ecosystem services destroyed by deforestation exceeds the economic benefits of agriculture, except in a few regions if greater yields of high-value crops are eventually realised. Globally, the numbers involved should grab the attention of policymakers. When forest was replaced with crops already present within individual countries, the mean net value of agriculture was either 75 or 103 billion international dollars less than the value of the ecosystem services that the original forests provided, depending on how carbon emissions were priced. Agriculture only delivered a net economic gain when all the crops that replaced forest were those that provided the economically highest returns for a given grid cell. However, this result comes from a somewhat unrealistic scenario, as it ignores local capacity for labour, knowledge, and infrastructure as well as price dynamics that alter agricultural production and international trade. For example, coffee is a highly profitable crop that would be planted across most of the tropics under the modelled scenario. However, the value of coffee would surely collapse if there was excess supply on global markets, and this was not considered. Closer work with macroeconomists is needed to redress this issue. Dynamical models that incorporate spatially explicit decision making by farmers within a global model of trade in agricultural commodities should produce more realistic outcomes [19].

Carrasco et al. [16] also help identify economically viable conservation targets. For example, deforestation for agriculture in South America, Indochina, the Philippines, and Madagascar was predicted to result in net economic losses because of the high-valued ecosystem services in these regions. Losses persisted even under future scenarios in which knowledge and infrastructure were available to plant the crops that generated the highest revenue for a given grid cell. By contrast, Southeast Asia was identified as an area where agricultural conversion was profitable, indicating that monetised conservation interventions such as REDD+ may need to be reconsidered and more tailored to local contexts.

## More effort needed to narrow uncertainty

**Fig 2:**
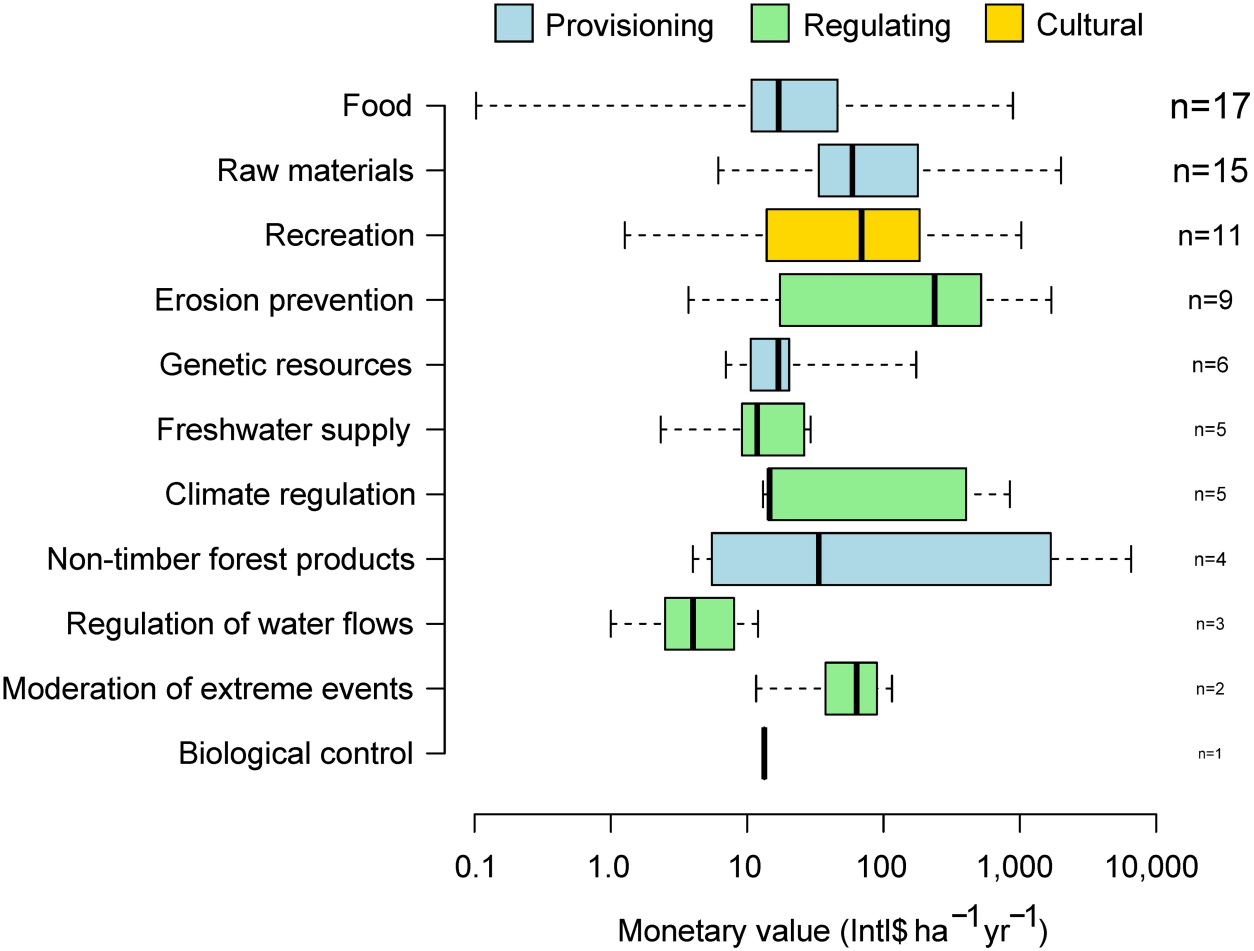
Monetary value of ecosystem services in tropical forests. Bars are medians, boxes are interquartile ranges, and whiskers are the full range of values for each of 11 ecosystem services. The monetary values compiled by Reference [15] were standardised into 2016 international dollars per hectare per year by Carrasco et al. [16] and are available therein as S1 Data. Ecosystem services were classified into 3 broader categories: provisioning, regulating, or cultural. *n* is the number of studies for each ecosystem service and is scaled proportionately to sample size.

Although the analysis of Carrasco et al. [16] is laudable, there remains large uncertainty in estimating the monetary values of ecosystem services. Future research can address this issue in at least 3 ways. Foremost, more data for individual ecosystem services should be generated and synthesised. In particular, there is a great opportunity to expand The Economics of Ecosystems and Biodiversity (TEEB) database [15], which underlies Carrasco et al. [16]. The publically available TEEB database only includes valuations until 2010, despite the thousands of ecosystem services studies published henceforth. Carrasco et al. [16] also find that only 78 observations from 30 studies are amenable to their modelling efforts. Better sampling effort should reduce the large variability in these observations and better account for cultural services, which are presently underrepresented (Fig 2). Second, sensitivity analyses can improve understanding of how different assumptions influence model predictions. One such sensitivity analysis might be to test different pricing structures for different ecosystem services. Analyses could be run by repeatedly sampling prices from probabilistic distributions defined by a mean and variance that summarise empirical studies, as in Fig 2. Carrasco et al. [16] use this approach for crop and carbon prices, and it could be extended for prices of other ecosystem services. Finally, more ecological and socioeconomic context could be added to valuation studies so as to improve meta-analytical models that predict monetary values in new sites. About half of the variation in the value of ecosystem services was unexplained in the models of Carrasco et al. [16]. This finding suggests that further effort might help narrow the large uncertainties in predicting ecosystem services, e.g., global valuations can vary by tens of billions of dollars [16].

Several other variables also remain to be considered in economic analyses of land-use decisions. The first is biodiversity. In the absence of valuation—a minefield in its own right—indices can be derived and targets set for goals such as no net biodiversity loss, e.g. [20]. However, biodiversity may trade off against both agricultural production and ecosystem services in forests. This is because both valuable ecosystem services and agricultural production typically require some proximity to human beneficiaries, whilst biodiversity is often higher further from people [21]. Consequently, factoring biodiversity into decision making might make regions such as Southeast Asia much less desirable for agricultural conversion given its high density of endemic species [16]. Second, agriculture is also a source of ecosystem services. For example, agricultural practices can enhance pollination, soil carbon sequestration, and cultural heritage [22]. As these benefits tend to be outweighed by the negative externalities generated by agriculture, analyses that ignore them are likely overestimating potential costs from land conversion.

## Implications

Conservations need not be dismayed at finding that it does not always pay to protect forests. There are certainly other reasons for conserving forests besides economic value, such as improving social equity, and a degraded forest can have value if there are no other forests for local people to use [23]. Therefore, monetary value is only one decision tool that policymakers should use alongside other, often local, evidence for determining conservation priorities. Carrasco et al. [16] have made considerable progress toward the challenge of delivering robust economic evidence and have highlighted where land clearance would be socially undesirable. Their analytical framework and results should inform the spatial prioritisation of real-world interventions such as REDD+. More broadly, identifying the spatial distribution of trade-offs in different land uses and their associated uncertainties can help deliver better environmental and economic outcomes for the whole planet.

## Abbreviations

REDD+: the United Nation’s initiative for Reducing Emissions from Deforestation and Forest Degradation
TEEB: The Economics of Ecosystems and Biodiversity
